# Perspectives on the Person-Centered Practice of Healthcare Professionals at an Inpatient Hospital Department: A Descriptive Study

**DOI:** 10.3390/ijerph20095635

**Published:** 2023-04-25

**Authors:** Diana Alves Vareta, Célia Oliveira, Carlos Família, Filipa Ventura

**Affiliations:** 1 PhD Program, University of Lisbon (UL) and Nursing School of Lisbon (ESEL), 1600-214 Lisboa, Portugal; 2 Egas Moniz Interdisciplinary Research Centre (CiiEM), Egas Moniz Universitary Institute, Quinta da Granja, 2829-511 Monte de Caparica, Portugal; 3 Nursing School of Lisbon (ESEL), 1600-096 Lisboa, Portugal; 4 Laboratory of Molecular Pathology and Forensic Biochemistry, Egas Moniz Universitary Institute, Quinta da Granja, 2829-511 Monte de Caparica, Portugal; 5 The Health Sciences Research Unit: Nursing (UICISA:E), Nursing School of Coimbra (ESEnfC), 3000-076 Coimbra, Portugal

**Keywords:** patient-centered care, person-centered practice inventory-staff, multidisciplinary care team, inpatient, noncommunicable diseases

## Abstract

The characteristics of health professionals and their understanding of person-centeredness may have important implications for the development of person-centered practice in specific care settings. In this study, we characterized the perceptions of the person-centered practice of a multidisciplinary team of health professionals working in the internal medicine inpatient unit of a Portuguese hospital. Data were collected using a brief sociodemographic and professional questionnaire and the person-centered practice inventory-staff (PCPI-S), and the effect of different sociodemographic and professional variables on each PCPI-S domain was determined using an analysis of variance (ANOVA). The results showed that a person-centered practice was positively perceived in the major constructs of *prerequisites* (M = 4.12; SD = 0.36), *the practice environment* (M = 3.50; SD = 0.48), and *person-centered process* (M = 4.08; SD = 0.62) domains. The highest scored construct was *developed interpersonal skills* (M = 4.35; SD = 0.47), and the lowest was *supportive organization systems* (M = 3.08; SD = 0.80). Gender was found to influence the perceptions of *knowing self* (F(2,75) = 3.67, *p* = 0.03, partial η^2^ = 0.089) and *the physical environment* (F(2,75) = 3.63, *p* = 0.03, partial η^2^ = 0.088), as was profession on *shared decision-making systems* (F(2,75) = 5.38, *p* < 0.01, partial η^2^ = 0.125) and *commitment to the job* (F(2,75) = 5.27, *p* < 0.01, partial η^2^ = 0.123), and the educational level on being *professionally competent* (F(1,75) = 4.99, *p* = 0.03, partial η^2^ = 0.062) and having *commitment to the job* (F(2,75) = 4.49, *p* = 0.04, partial η^2^ = 0.056). In addition, the PCPI-S proved to be a reliable instrument for characterizing healthcare professionals’ perceptions of the person-centeredness of care in this context. Identifying personal and professional variables that influence these perceptions could provide a starting point for defining strategies to move practice toward person-centeredness and for monitoring changes in healthcare practice.

## 1. Introduction

Person-centered care is increasingly recognized as a fundamental approach to the quality, safety, and sustainability of health services, and is anchoring a new paradigm in the international development of health policies and strategies [1,2,3].

Person-centered care integrates the perspectives of individuals, families, and communities as users and participants, enabling us as healthcare professionals to meet the needs of the population across the lifespan [3]. It is defined as “…an approach to practice established by shaping and promoting healthy relationships between carers, service users, and people significant to their lives. It is underpinned by values of respect for people, individual right to self-determination, mutual respect, and understanding. It is enabled by cultures of empowerment that promote continuous approaches to practice development” [4].

Along with the recognition of the importance of establishing integrated and person-centered health services [3], several global challenges for their implementation in clinical practice have been identified [4,5,6,7]. Contextual factors, such as the organizational culture, the characteristics of health professionals, and the practice environment, pose the greatest challenges to developing cultures that support person-centered care [8,9,10,11]. According to the WHO [3], each country should define a strategy that considers the specificities of its health system, care setting, and health professionals.

The development of the person-centered practice framework (PCPF) [4,5] and of the instruments to monitor its presence in clinical practice [10] has guided its implementation in healthcare settings to date. The PCPF presents the critical domains and concepts inherent to person-centered care and provides a reference for its implementation and development [4,5]. The person-centered practice inventory is an instrument that maps to the theoretical domains of the PCPF and allows for an understanding of its practice, the identification of areas of potential improvements, and the design of targeted interventions to enhance person-centeredness [12].

How healthcare professionals think about and value person-centeredness in care can have important implications for how they construct their decisions and represent their actions in practice [13]. Several studies have highlighted the need for health professionals to orient the focus of care towards a person’s values, thoughts, and experiences in order to be more person-centered, rather than simply adhering to pre-established norms [14,15,16,17]. However, few studies have focused on the perceptions of healthcare professionals in multidisciplinary teams regarding person-centered care in the hospital setting [18,19,20].

This study is part of a clinical study protocol [21] designed to provide recommendations for the development of person-centered practice in the daily care of hospitalized older adults with chronic diseases within an internal medicine department. The current practice analysis refers to how person-centered practice is perceived and identified in the context under study, considering that all principles and domains presented in the PCPF are fundamental for the implementation of this practice. Therefore, the present study aims to characterize the perceptions of person-centered practice conducted by healthcare professionals whom comprise the multidisciplinary team of an inpatient hospital unit and explore the influence of sociodemographic and professional variables on their perceptions.

## 2. Materials and Methods

### 2.1. Design and Population

A quantitative, descriptive, cross-sectional approach was adopted in this study. It was carried out in an internal medicine inpatient unit in a secondary hospital located in an urban area in Portugal, with a direct impact area of 500,000 inhabitants. The internal medicine unit had a capacity of 55 inpatient beds. The multidisciplinary team comprises the medical staff, including 4 senior graduate physicians, 12 graduate physicians, 18 physicians, and 20 specific-training interns; the nursing staff, consisting of 50 general care nurses and 3 specialist nurses; and the physiotherapy staff, containing 2 physiotherapists. As long as they met the inclusion criteria, all physicians, nurses, and physiotherapists working in the unit were eligible for inclusion in the study sample.

### 2.2. Inclusion Criteria

All healthcare professionals working full-time in the internal medicine unit for the last six months were eligible to participate in the study. This ensured that all healthcare professionals in the multidisciplinary team perspectives on person-centered practice in the setting were covered.

### 2.3. Exclusion Criteria

Healthcare professionals from other departments or specialties who provided care on an occasional basis in the context of the study were excluded.

### 2.4. Data Collection

Data were collected between December 2021 and January 2022. A questionnaire including a section for the sociodemographic characterization (Table 1) and the person-centered practice inventory-staff (PCPI-S) [10,22] was provided to the healthcare professionals who met the defined criteria.

The PCPI-S is a valid scale, psychometrically accepted and validated by an international panel of experts in person-centered practice [10,23], with proven reliability [10,12,24,25,26]. It enabled us to understand how health professionals perceived their practice concerning person-centredness. The PCPI-S is a self-report instrument composed of 59 items on a five-point Likert-type response scale, with higher scores indicating a greater agreement [10]. The PCPI-S measures three domains derived from the PCPF (i.e., *prerequisites, the practice environment,* and *person-centered processes*) and comprises 17 constructs, including *professionally competent, having developed interpersonal skills, knowing self, clarity of beliefs and values, commitment to the job, appropriate skill mix, shared decision-making systems, effective staff relationships, power sharing, the physical environment, supportive organizational systems, the potential for innovation and risk-taking, working with the person’s beliefs and values, sharing decision making, engaging authentically, being sympathetically present,* and *working holistically* (Table 2) [5].

The PCPI-S was translated and culturally adapted into Portuguese and showed acceptable psychometric properties and good reliability [22]. 

The questionnaire was delivered to the healthcare professionals using an institutional email via Google Forms^®^ (Google Corp. 2018, Dublin, Ireland) to ensure the anonymity of the data collected. Healthcare professionals were encouraged to participate during the period available for data collection by sending a reminder email one week before the end and extending the response period by two weeks.

### 2.5. Statistical Data Analysis

Quantitative data were analyzed using a statistical package for social sciences software (IBM SPSS Statistics^®^ for Windows, v. 27.0. IBM Corp. Released 2020, Armonk, NY, USA). A descriptive analysis (i.e., mean, standard deviation, minimum, and maximum) of the constructs comprising the PCPI-S was performed. An analysis of variance (ANOVA) was then performed to determine the effect of demographic and professional variables on the PCPI-S constructs, as described in the previous section. Specifically, dependent variables with more than two response options, for which statistically significant differences were found, were further evaluated using Tukey’s post hoc test for multiple comparisons. Model assumptions were assessed by analyzing a plot of residuals versus predicted values, a Q–Q plot of residuals, and a residual histogram. Whenever a violation of the ANOVA assumptions occurred, the dependent variable or construct was transformed using a Box–Cox transformation. A new ANOVA was then run, and the assumptions were reverified as described above.

The plot of residuals versus predicted values, the Q–Q plot of residuals, and a residual histogram were generated in the R language statistical computing v.4.2.2 [27] using the resid_auxpanel function from the ggResidpanel v.0.3.0 library [28] after importing the SPSS data file containing the predicted and residual values of each ANOVA model using the read.spss function from the foreign v.0.8-83 library [29].

A *p*-value of <0.05 was considered for statistical significance [10,12,25] and values were rounded to the nearest hundredth.

### 2.6. Ethical Considerations

The study received ethical approval from the ethics committee of the hospital where the study took place (ref. nr. 36/2021). All procedures were performed in accordance with the Declaration of Helsinki [30] and in compliance with the General Data Protection Regulation [31]. Permission to use the PCPI-S was requested and granted by the authors.

## 3. Results

### 3.1. Characteristics of the Participants

The convenience sample included 109 health professionals, with a response rate of 76.15% (N = 83). The sample was predominantly composed of female professionals (79.5%), and the most representative professional group was nurses (61.5%), followed by physicians (36.1%) and physiotherapists (2.4%). Regarding the educational level, 62.7% of the health professionals had a university degree and a maximum of 24 years of education (M = 17.6; SD = 2.09). The sample had a mean of 11 years of professional experience (SD = 8.06; Min = 0; Max = 40), with 45.8% of the healthcare professionals having less than 10 years of experience, 41% having between 10 and 20 years, and 13.3% having more than 20 years of experience (Table 1).

### 3.2. Perception of Person-Centered Practice

The results were analyzed using the mean score of the response scale (one to five points), accordingly to the authors’ guidelines [9]. Constructs with a mean score greater than 2.5 were considered positive, indicating an agreement among healthcare professionals.

All domains were positively rated by the different professional groups (Table 2). The *prerequisites* domain had the highest score (M = 4.12; SD = 0.36), followed by *person-centered processes* (M = 4.08; SD = 0.51) and *the practice environment* (M = 3.50; SD = 0.48).

Three constructs with very high scores emerged from the *prerequisites* domain, namely, *developed interpersonal skills* (M = 4.35; SD = 0.47), which had the highest score of all the constructs analyzed, *professionally competent* (M = 4.26; SD = 0.42), and *commitment to the job* (M = 4.25; SD = 0.42). Conversely, *clarity of beliefs and values* (M = 3.66; SD = 0.60) and *knowing self* (M = 3.91; SD = 0.72) had the lowest scores.

In *the practice environment*, the highest scoring construct was *appropriate skill mix* (M = 4.02; SD = 0.52), and the lowest scoring was *supportive organization systems* (M = 3.08; SD = 0.80), which represented the lowest score of all 17 constructs.

In *person-centered processes*, *working holistically* (M = 4.22; SD = 0.62) and *engaging authentically* (M = 4.17; SD = 0.52) had the highest response scores, and *sharing decision making* (M = 3.91; SD = 0.72) had the lowest.

### 3.3. Influence of Sociodemographic and Professional Characteristics

#### 3.3.1. Prerequisites

Gender, profession, and educational level were found to have a significant effect on the constructs of the *prerequisites* domain.

The educational level significantly influenced the health professionals’ perceptions of being *professionally competent* (F(1,83) = 4.98, *p*-value = 0.029, partial η^2^= 0.062) (Appendix A.1), with a decrease in the value assigned to *professionally competent* of 0.218 between professionals with and without a degree.

Participants’ professions also significantly influenced the perceptions of *commitment to the job* (F(2,83) = 5.27, *p*-value = 0.007, partial η^2^= 0.123) (Appendix A.2). There were significant differences between the perceptions of physicians (M = 4.25; SD = 0.43) and nurses (M = 4.22; SD = 0.39) when compared to physical therapists (M = 5; SD = 0.0).

In addition, the educational level significantly influenced the construct of *commitment to the job* (F(1,83) = 4.49, *p*-value = 0.037, partial η^2^= 0.056) (Appendix A.2), indicating that there were significant differences between professionals who held a degree (M = 4.19; SD = 0.42) compared to those who completed postgraduate studies (M = 4.35; SD = 0.40).

Participants’ genders significantly influenced *knowing self* ‘(F(2,83) = 3.67, *p*-value = 0.030, partial η^2^= 0.089) (Appendix A.3), where being female increased the perception of *knowing self* (B = 0.527).

No significant effect due to the independent variables was found in the constructs *clarity of beliefs and values* (Appendix A.4) nor *developed interpersonal skills* (Appendix A.5).

#### 3.3.2. The Practice Environment

The profession and gender of healthcare professionals were found to significantly influence perceptions of *the practice environment* domain. In addition, profession was found to significantly influence *shared decision-making systems* (F(2,83) = 5.38, *p*-value = 0.007, partial η^2^= 0.125) (Appendix A.6). There were significant differences in the perceptions of physicians (M = 3.60; SD = 0.63) compared to nurses (M = 3.08; SD = 0.84) and physiotherapists (M = 2.75; SD = 1.06).

Gender significantly influenced *the physical environment* (F(2,83) = 3.63, *p*-value = 0.031, partial η^2^= 0.088) (Appendix A.7), with women having a higher score assigned to the perception of *the physical environment* (B = 0.532).

No significant effect due to the independent variables was found in the *appropriate skill mix* (Appendix A.8), *effective staff relationships* (Appendix A.9), *power sharing* (Appendix A.10), the *potential for innovation and risk-taking* (Appendix A.11), and *supportive organization systems* (Appendix A.12).

#### 3.3.3. Person-Centered Processes

No significant effect due to the independent variables was found for any of the constructs in the *person-centered processes* domain, namely, *working with the person’s beliefs and values* (Appendix A.13), *sharing decision making* (Appendix A.14), *engaging authentically* (Appendix A.15), *being sympathetically present* (Appendix A.17), and *working holistically* (Appendix A.16).

### 3.4. Reliability of the PCPI-S

All domains had an α of >0.85, and there was a significant correlation between them (Appendix B). The internal consistency of each domain of the PCPI-S was assessed, with *prerequisites* and *the practice environment* showing good consistency (α = 0.85 and α = 0.88, respectively) and *person-centered processes* showing excellent consistency (α = 0.94) (Table A18) [32]. Overall, an adequate internal consistency of the inventory was found when applied to the study sample.

## 4. Discussion

The sample reflected the Portuguese reality, where 76.5% of healthcare workers were female and the most represented professional group were nurses [33]. According to the Organization for Economic Cooperation and Development [2], the nurse/physician ratio is 1.3, whereas in the sample of this study, the ratio was 1.7.

Healthcare professionals’ perceptions of person-centered practice were positive, with all constructs having mean scores greater than 2.5 (min = 3.08; max = 4.35).

The *prerequisites* related to the characteristics of the multidisciplinary team and were considered the critical foundations for the development of professionals toward a person-centered practice [4,5]. Healthcare professionals valued the *prerequisites* of *developed interpersonal skills* (M = 4.26; SD = 0.42), *professionally competent* (M = 4.35; SD = 0.47), and *commitment to the job* (M = 4.25; SD = 0.42), giving relevance to communicating effectively, demonstrating commitment to finding mutual solutions, and providing holistic care that integrates knowledge, skills, and experience to negotiate care options [4,5].

However, *clarity of beliefs and values* received the lowest score of the domain (M = 3.66; SD = 0.60). This construct related to the awareness of the impact of professionals’ beliefs and values on the care provided and the commitment to reconcile them to facilitate person-centeredness [4,5]. Similarly, *knowing self* was related to self-awareness and to the perception of the person regarding self-knowledge, which, although showing a positive score, was below four (M = 3.91; SD = 0.72). This result may be related to the lack of critical thinking and a reflection of the practice in the study context, as both constructs depended on individual development based on reflection. McCance et al. [23] reiterated that *clarity of beliefs and values* of healthcare professionals is the foundation for culture changes, which are essential for professionals to move towards person-centeredness.

Participants in the study valued the interpersonal relationships and their commitment and involvement in professional practice, having the potential to develop the team’s shared professional values and demonstrate them in practice. Aligning the values adopted by the team with the behaviors experienced in practice is essential to transform the culture, context, and consistency of the care provided [5,34].

When comparing the results of this study with studies using a similar methodology, in which the PCPI-S was applied to nurses in a hospital setting, such as the study by Slater et al. [12], similar results were obtained for each construct, where *commitment to the job* (M = 4.45; SD = 0.40), *professionally competent* (M = 4.26; SD = 0.41), and *developed interpersonal skills* (M = 4.37; SD = 0.38) scored highest, whilst *clarity of beliefs and values* (M = 3.91; SD = 0.54) and *knowing self* (M = 4.04; SD = 0.52) scored lowest. Tiainen et al. [25] also obtained similar results for *developed interpersonal skills* (M = 4.08; SD = 0.48) and being *professionally competent* (M = 4.07; SD = 0.51). McCance et al. [23] conducted a study with a multidisciplinary sample that also highlighted the *commitment to the job* (M = 4.39; SD = 0.47), *professionally competent* (M = 4.24; SD = 0.46), and *developed interpersonal skills* (M = 4.32; SD = 0.43) as the most valued constructs, and *clarity of beliefs and values* (M = 3.90; SD = 0.58) and *knowing self* (M = 3.96; SD = 0.58) as the least valued.

The *prerequisites* domain is essential in triggering significant changes in *the practice environment* and the professionals’ involvement in *person-centered processes* [23,35]. As this domain was the most valued by health professionals, it suggested the existence of individual conditions for the development of person-centered practice in context.

*The practice environment* domain refers to contextual aspects and influences the operationalization of person-centered practice through its potential to facilitate or inhibit *person-centered processes* [4,5]. Herein, the constructs that showed lower mean scores belonged to the domains of *prerequisites* and *person-centered processes*, with results that were similar to those obtained by Slater et al. [12], Tiainen et al. [25], and McCance et al. [23]. In addition, Johnsen et al. [20] reported that healthcare professionals working in acute inpatient hospital settings identified fewer aspects of organizational culture related to person-centered practice, reinforcing the need to emphasize the environmental aspects in this context.

In *the practice environment*, the health professionals in the sample rated the multidisciplinary team’s knowledge and skill mix as essential to providing quality care, scoring high in *appropriate skill mix* (M = 4.02; SD = 0.52). The studies conducted by Slater et al. [12], Tiainen et al. [25], and McCance et al. [23] also showed an *appropriate skill mix* with the highest scores (M = 4.22, SD = 0.45; M = 4.15, SD = 0.46 and M = 4.15, SD = 0.51, respectively).

The lowest scoring constructs referred to *supportive organization systems* (M = 3.08; SD = 0.80), i.e., organizational systems that promote people’s initiative, creativity, freedom, and security, supported by a structure that privileges culture, relationships, values, communication, professional autonomy, and accountability [4,5]. Of the 17 constructs analyzed, *supportive organization systems* received the lowest score, indicating that professionals perceived a lack of support from the organization in areas critical to practice changes. To better understand these results, it would be important to characterize the environment of care and the institution’s mission, values, and regulations to determine whether these aspects are consistent with or supportive of person-centered practice. McCance et al. [36] and Hower et al. [35] identified the significant impact of contextual factors on the implementation of person-centered practice. They recognized the importance of the institution in changing practice and promoting a person-centered culture. Slater et al. [12], Tiainen et al. [25], and McCance et al. [23] also found identical results for these constructs (M = 3.43, SD = 0.66; M = 3.25; SD = 0.48 and M = 3.18, SD = 0.83, respectively).

With a similarly low score, the *shared decision-making systems* construct (M = 3.26; sd = 0.81) refers to the organizational commitment to collaborative and participatory ways in which the healthcare team engages in decision making. McCance et al. [23] also found this construct to be a predictor of person-centered culture, reinforcing the importance of interdisciplinarity and patient involvement in care. The low perception of *shared decision-making systems* in our study may indicate that healthcare professionals need to be committed to a collaborative culture that involves all participants in decision making. Otherwise, the patient’s involvement in care may be compromised.

*Person-centered processes* describe care delivery, operationalized through a set of person-centered activities [5]. Here, healthcare professionals scored highest on *working holistically* (M = 4.22, SD = 0.62), representing their value in integrating physiological, psychological, sociocultural, developmental, and spiritual dimensions into care delivery. Similarly, the scores on *engaging authentically* (M = 4.17, SD = 0.52) highlighted the recognition of the importance of the professional’s connection to the person being cared for and the people who matter to them, as determined by the knowledge of the person, clarity of their beliefs and values, knowing self, and professional experience [4,5].

In the studies by Slater et al. [12], Tiainen et al. [25], and McCance et al. [23], a higher score was also found in *working holistically* (M = 4.40, SD = 0.44; M = 4.14, SD = 0.55 and M = 4.30, SD = 0.53, respectively). In *engaging authentically*, similar scores were obtained in the referred studies (M = 4.18, SD = 0.46; M = 4.01, SD = 0.47 and M = 4.20, SD = 0.47, respectively), although it was not the most valued construct within them. The lowest scores were assigned to *being sympathetically present* (M = 4.07, SD = 0.59), *working with the person’s beliefs and values* (M = 4.05; SD = 0.52), and the *sharing decision making* (M = 3.91; SD = 0.72) constructs.

McCance et al. [23] suggested that *being sympathetically present* is a core element concerning all of the other *person-centered processes* constructs, and is highly connected with *working with the person’s beliefs and values* as it depends on knowing the patients and having insight into their beliefs and values to maximize coping resources.

The construct of *sharing decision making* (M = 3.91; SD = 0.72) showed the lowest response score within the *person-centered processes*, revealing that healthcare professionals should recognize their role in facilitating and reinforcing the patient’s involvement in decision making [4,5]. This construct was also closely linked to *working with the person’s beliefs and values* as the foundation of the involvement in decision making are sustained on the person’s values, experiences, concerns, beliefs, and future aspirations. Knowing that *working with the person’s beliefs and values* supports and influences these structural constructs could be a key to the development of person-centered practice in this context.

The low score obtained in *sharing decision making* was not surprising when verifying the score of *shared decision-making systems*. Without an organizational commitment shared among healthcare professionals, the team cannot engage with the patient in decision making [4].

In the study of Gregório et al. [37], which was conducted in a representative sample of the Portuguese population, most people preferred a controlling role of the professional rather than actively participating in clinical decision making. Healthcare professionals should be alert to this fact and increasingly recognize the importance of the person’s involvement in clinical decision making for person-centeredness. Tiainen et al. [25] had similar results (M = 3.92, SD = 0.53) in *sharing decision making*. However, in the studies by Slater et al. [12] and McCance et al. [23], the score was higher (M = 4.21, SD = 0.52 and M = 4.09, SD = 0.58, respectively).

The consistency of the results in the *prerequisites* and *the practice environment* with studies of similar characteristics may be related to the fact that all studies were conducted in Europe, namely, in England [12], Finland [25], and Ireland [23]. The cultural similarity could have influenced the characteristics of both the practitioners and the contexts.

Concerning the influence of sociodemographic and professional characteristics on the perception of person-centered practice, the female gender positively influenced the constructs of *knowing self* and *the physical environment*. In healthcare professions, women tend to be prevalent. Therefore, understanding the influence of gender on the care provided is essential.

The construct of *knowing self* refers to how the healthcare professional gives meaning to knowledge and action, using reflection, self-awareness, and engagement with others in the search for a person-centered practice [4,5]. Al-surimi et al. [38] justified the difference in this perception, as female professionals naturally value the relational aspects of care. An aesthetically pleasant physical environment stimulates the senses and promotes healing, well-being, care, and involvement in interprofessional relationships [4,5]. Female professionals value this aspect, while males may tend to focus more on the interventions and procedural aspects of care rather than on the characteristics of the physical environment [38].

The profession significantly influenced the constructs of *shared decision-making systems* and *commitment to the job*. Gemmae et al. [39] and Dahlke et al. [40] reported that professional groups had different perceptions about the fundamental concepts of person-centered practice according to their intervention area. Physicians had a more positive perception of the *shared decision-making systems* than nurses and physiotherapists. Professional interdependence and the degree of autonomy of each professional group may explain this result. However, this construct was the least valued by the multidisciplinary team (M = 3.26; SD = 0.81), indicating the need to strengthen the commitment to participatory collaboration between team members and patients. *Shared decision-making systems* are a likely predictor of the development of a person-centered culture due to the importance of shared decision making among the multidisciplinary team and the person’s involvement in care [22]. Given the results obtained in this construct, it could be expectable that the profession would exert a similar influence on the constructs of *power sharing* and *effective staff relationships*, which did not occur. These results could indicate that despite recognizing the absence of *shared decision-making systems* among professionals and patients, the study participants perceived the relationships in the team and *power sharing* as favorable to a person-centered practice. This relationship should be the focus of qualitative inquiry if the aim is to improve the quality of care toward person-centeredness.

Concerning *commitment to the job*, physiotherapists were assigned a higher score than physicians and nurses. Commitment to persons and family through the professionals’ involvement in the relationship was valued by those who spent less time in contact with patients. However, this construct should be analyzed in a broader spectrum since commitment as a multidisciplinary team member should overlap with individual commitments [4,5]. Thus, the discrepancy in perceptions between the different healthcare professionals in this study could reveal the absence of a shared commitment at the organizational level. In addition, the length of training showed an increasing influence on the *commitment to the job*, which was not surprising considering that engagement in the relationship is supported by a holistic view based on evidence and education [4,5].

The educational level also influenced the construct of *professionally competent*. Professionals with higher education tend to value knowledge, skills, and attitudes for negotiating care options [4,5]. The *professionally competent* aspect includes professional knowledge and experience. However, professional experience did not influence this construct in the sample studied.

Professional experience did not influence the perception of any construct. This was in contrast to the study by Esmaeili et al. [19], which showed that professional experience was associated with the provision of holistic, collaborative, and comprehensive care. Similarly, the study by Tiainen et al. [24] showed a positive influence of nurses’ professional experiences on the perception of the constructs of *professionally competent* and *the physical environment*. Johnsen et al. [20] found that health professionals with postgraduate education showed greater involvement to patients in decision making than those with a degree. The fact that professional experience did not influence the perception of person-centeredness in this study may be related to the categorization of the variable. The categorization was determined to facilitate comparisons with previous studies that used the same methodology. However, the categorization may need to be reviewed.

The statistically significant differences on the scores of the constructs between the different groups highlighted the usefulness of the PCPI-S in identifying areas of development that are appropriate for different professionals.

Overall, healthcare professionals in the context studied demonstrated an understanding of person-centered practice in their work context.

In summary, in *prerequisites*, the construct of *clarity of beliefs and values* revealed the need to gain an awareness of its impact on the healthcare experience and the need to develop team-aligned values to move toward a person-centered culture. *The practice environment* was identified as the domain requiring greatest investment with lower scores on the PCPI-S. The *supportive organization systems* and *shared decision-making systems* indicated the lack of organizational systems that promoted professional initiative, creativity, and autonomy, and ones that value communication, relationships, and participation among healthcare professionals. The low score for the *shared decision-making* main theme was reinforced in the *person-centered processes*, highlighting the need for healthcare professionals to be reinforced as facilitators of participation in the setting and to work on recognizing the person’s values, experiences, concerns, and beliefs, as their individual perspective and psychosocial role are the foundation of decision making.

Therefore, these aspects should be the focus of special attention to improve person-centeredness. In order to initiate and sustain an effective change toward person-centered practice, its components must also be identified at all levels of care delivery [4,5]. Therefore, in addition to aligning all levels of care with the principles of person-centered practice, it is necessary to ensure that aspects of *the practice environment* are sufficiently valued in the context [36]. McCormack et al. [8] suggested that contextual factors, such as the organizational culture, the learning environment, and the care environment itself, pose the greatest challenge to person-centeredness and the development of cultures that can support person-centered practice.

## 5. Limitations

This study was one of the first to systematically assess the factors influencing person-centeredness in a multidisciplinary team in a hospital setting, which limited the comparability of the results.

The categorization of the sociodemographic variables was chosen in order to allow for a comparison with studies with similar methodologies. However, this may have limited us from conducting a more in-depth analysis of the impact of the variable on the healthcare professionals’ perception of person-centered care.

The high scores obtained on the different constructs raised the question of whether the participants’ responses reflected their idealized practice or their real and current perception of daily care. Therefore, qualitative studies with multidisciplinary samples should be conducted to triangulate the results obtained in this study, as suggested by Vareta et al. [21].

The fact that the research was conducted in a specific context, namely, in an internal medicine department with a small sample of healthcare professionals, limited the possibility of transferability to other care settings or populations. However, this involved a multidisciplinary team with a high response rate, which was considered a strength.

## 6. Conclusions

The key concepts for implementing and developing person-centered practice in inpatient settings were positively identified by the professionals of the multidisciplinary team of the study context.

The PCPI-S proved to be sensitive in identifying health professionals’ perceptions of the person-centeredness of their practice and in identifying significant differences in perceptions between groups, taking into account their personal and professional characteristics and, thus, contributing to the development of care practice. In addition, the influence of sociodemographic and professional characteristics on the scores obtained, considered statistically significant, allowed for the identification of groups and areas of differentiated intervention for sustainable practice developments specifically adapted to the context and person.

The results of this study contributed to a growing evidence base regarding the PCPI as a psychometrically sound instrument that allows for the structural concepts of an established theory to be identified and inform changes in practice.

Characterizing the culture and organizational structure of the context in future studies could allow for a deeper understanding of the relationships between the domains and the multifaceted factors that facilitate or limit them. It would be essential to analyze the influence of the values and customs of different countries on the perception of the person-centered practice.

## Figures and Tables

**Table 1:** Sociodemographic characteristics of the participants.

Sociodemographic Variables	n	%
Gender		
Prefer not to declare	1	1.2
Female	66	79.5
Male	16	19.3
Profession		
Nurse	51	61.5
Physician	30	36.1
Physiotherapist	2	2.4
Educational level		
Degree	52	62.7
Postgraduate studies	31	37.3
Professional experience		
<10 years	38	45.8
10–19 years	34	41
20–40 years	11	13.2

**Table 2:** Mean and Cronbach alpha scores of PCPF constructs [10].

Constructs	Mean (SD)	Cronbach α
Prerequisites	4.12 (0.36)	0.85
Professionally competent	4.26 (0.42)	0.45
Developed interpersonal skills	4.35 (0.47)	0.76
Commitment to the job	4.25 (0.42)	0.69
Knowing self	3.91 (0.72)	0.85
Clarity of beliefs and values	3.66 (0.60)	0.58
The practice environment	3.50 (0.48)	0.88
Appropriate skill mix	4.02 (0.52)	0.59
Shared decision-making systems	3.26 (0.81)	0.77
Effective staff relationships	3.62 (0.76)	0.75
Power sharing	3.51 (0.66)	0.71
Potential for innovation and risk-taking	3.59 (0.64)	0.68
The physical environment	3.74 (0.73)	0.80
Supportive organization systems	3.08 (0.80)	0.82
Person-centered processes	4.08 (0.51)	0.94
Working with the person’s beliefs and values	4.05 (0.56)	0.75
Sharing decision making	3.91 (0.72)	0.81
Engaging authentically	4.17 (0.52)	0.80
Being sympathetically present	4.07 (0.59)	0.81
Working holistically	4.22 (0.62)	0.84

**Figure A1:**
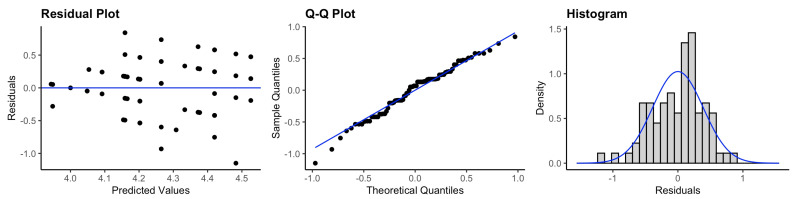
Plot of the Residuals vs. predicted value (**left**), Q–Q plot of residuals (**middle**), and histogram of residuals (**right**).

**Figure A2:**
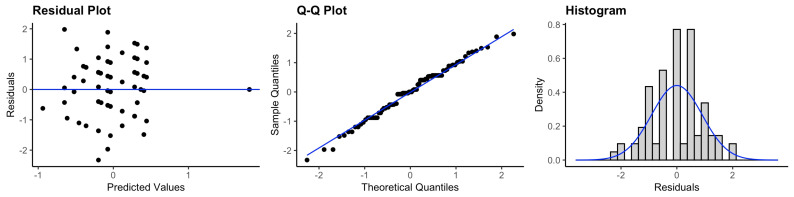
Plot of the Residuals vs. predicted value (**left**), Q–Q plot of residuals (**middle**), and histogram of residuals (**right**).

**Figure A3:**
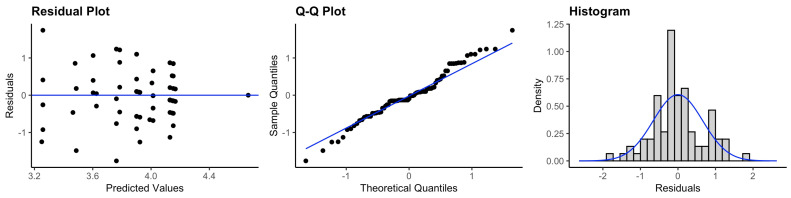
Plot of the Residuals vs. predicted value (**left**), Q–Q plot of residuals (**middle**), and histogram of residuals (**right**).

**Figure A4:**
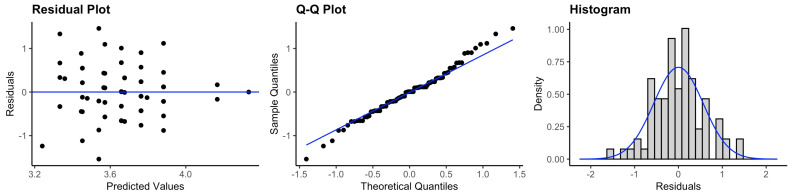
Plot of the Residuals vs. predicted value (**left**), Q–Q plot of residuals (**middle**), and histogram of residuals (**right**).

**Figure A5:**
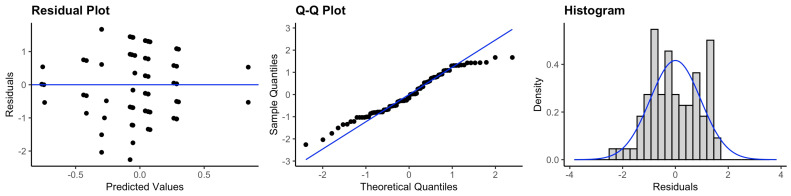
Plot of the Residuals vs. predicted value (**left**), Q–Q plot of residuals (**middle**), and histogram of residuals (**right**).

**Figure A6:**
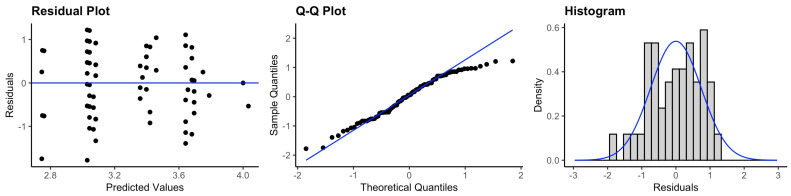
Plot of the Residuals vs. predicted value (**left**), Q–Q plot of residuals (**middle**), and histogram of residuals (**right**).

**Figure A7:**
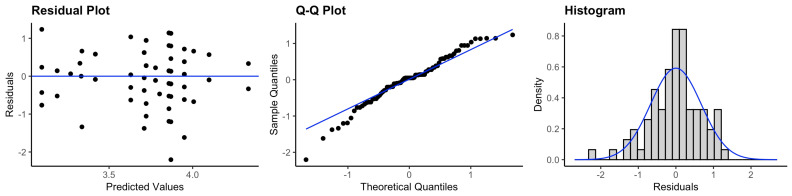
Plot of the Residuals vs. predicted value (**left**), Q–Q plot of residuals (**middle**), and histogram of residuals (**right**).

**Figure A8:**
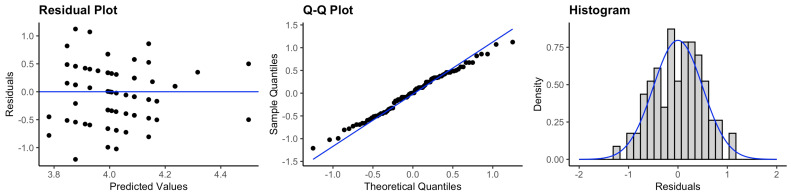
Plot of the Residuals vs. predicted value (**left**), Q–Q plot of residuals (**middle**), and histogram of residuals (**right**).

**Figure A9:**
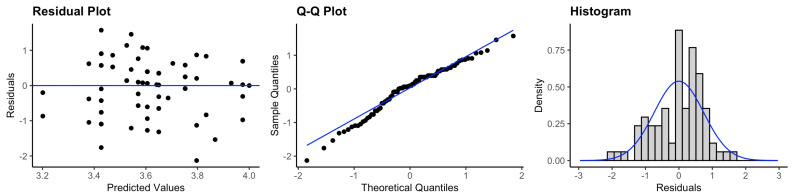
Plot of the Residuals vs. predicted value (**left**), Q–Q plot of residuals (**middle**), and histogram of residuals (**right**).

**Figure A10:**
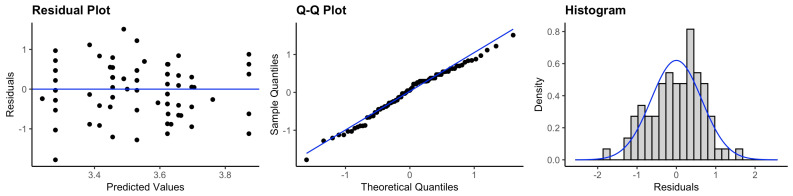
Plot of the Residuals vs. predicted value (**left**), Q–Q plot of residuals (**middle**), and histogram of residuals (**right**).

**Figure A11:**
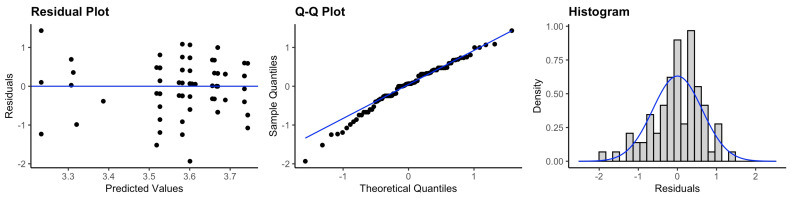
Plot of the Residuals vs. predicted value (**left**), Q–Q plot of residuals (**middle**), and histogram of residuals (**right**).

**Figure A12:**
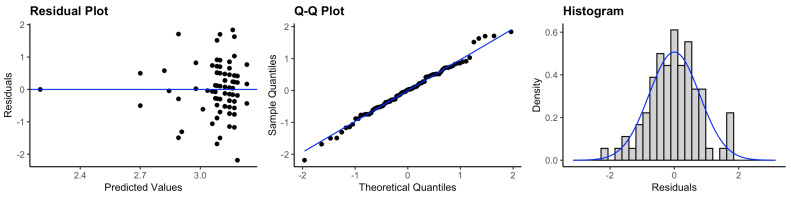
Plot of the Residuals vs. predicted value (**left**), Q–Q plot of residuals (**middle**), and histogram of residuals (**right**).

**Figure A13:**
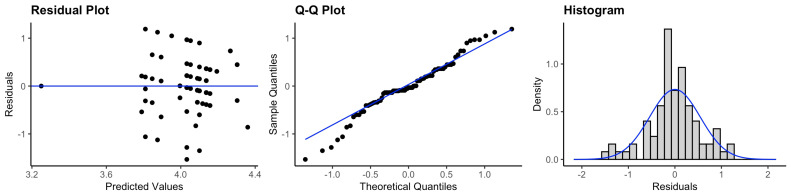
Plot of the Residuals vs. predicted value (**left**), Q–Q plot of residuals (**middle**), and histogram of residuals (**right**).

**Figure A14:**
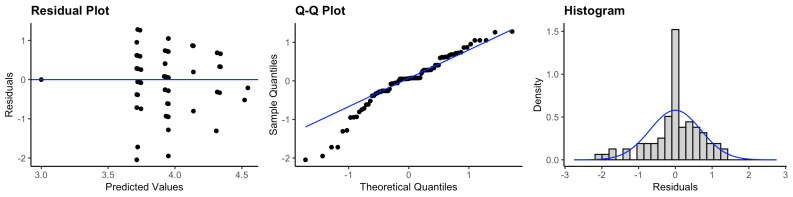
Plot of the Residuals vs. predicted value (**left**), Q–Q plot of residuals (**middle**), and histogram of residuals (**right**).

**Figure A15:**
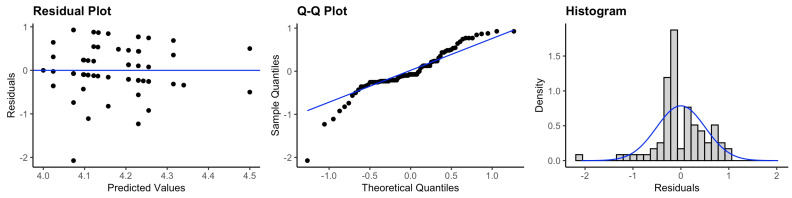
Plot of the Residuals vs. predicted value (**left**), Q–Q plot of residuals (**middle**), and histogram of residuals (**right**).

**Figure A16:**
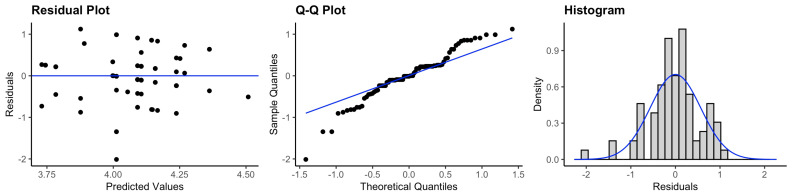
Plot of the Residuals vs. predicted value (**left**), Q–Q plot of residuals (**middle**), and histogram of residuals (**right**).

**Figure A17:**
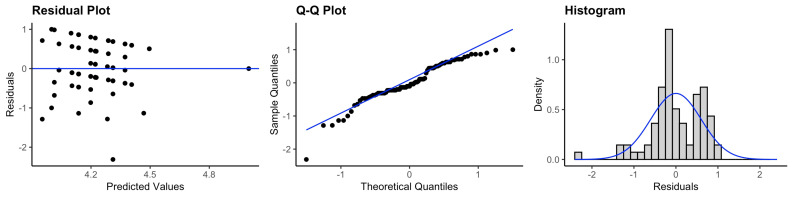
Plot of the Residuals vs. predicted value (**left**), Q–Q plot of residuals (**middle**), and histogram of residuals (**right**).

## Data Availability

Not applicable.

## Appendix A

### Appendix A.1. Professionally Competent

An analysis of variance (ANOVA) was performed to determine the effect of variables including gender, profession, educational level, and professional experience on the construct of *professionally competent* (Table A1). The analysis of the standardized residual diagnostic charts, residual Q–Q chart, and residual histogram (Figure A1) did not show any apparent violation of the model’s assumptions of normality and homoscedasticity.

**Table A1:** Results of the ANOVA model of the *professionally competent* construct. *p*-values of less than the 0.05 significance level were highlighted in bold.

Predictor	Sum of Squares	d*f*	Mean Square	*F*	*p*-Value	Partial η^2^
(Intercept)	1504.15	1	1504.150	9078.201	0.000	0.992
Gender	0.142	2	0.071	0.430	0.652	0.011
Profession	0.370	2	0.185	1.117	0.333	0.029
Educational level	0.826	1	0.826	4.987	**0.029**	0.062
Professional experience	0.413	2	0.207	1.247	0.293	0.032
Error	12.427	75	0.166			

### Appendix A.2. Commitment to the Job

An analysis of variance (ANOVA) was performed to determine the effect of gender, profession, educational level, and professional experience on the construct *commitment to the job* (Table A2).

The analysis of the standardized residual diagnostic charts, residual Q–Q chart, and residual histogram (Figure A2) did not show any apparent violation of the model’s assumptions of normality and homoscedasticity.

**Table A2:** Results of the ANOVA model of the *commitment to the job* construct. *p*-values of less than the 0.05 significance level were highlighted in bold.

Predictor	Sum of Squares	d*f*	Mean Square	*F*	*p*-Value	Partial η^2^
(Intercept)	1497.913	1	1497.913	9.677.33	0.000	0.992
Gender	0.379	2	0.190	1.225	0.300	0.032
Profession	1.631	2	0.816	5.270	**0.007**	0.123
Educational level	0.694	1	0.694	4.487	**0.037**	0.056
Professional experience	0.373	2	0.186	1.204	0.306	0.031
Error	11.609	75	0.155			

### Appendix A.3. Knowing self

An analysis of variance (ANOVA) was performed to determine the effect of the variables gender, profession, educational level, and professional experience on the construct of *knowing self* (Table A3).

The analysis of the standardized residual diagnostic charts, residual Q–Q chart, and residual histogram (Figure A3) did not show any apparent violation of the model’s assumptions of normality and homoscedasticity.

**Table A3:** Results of the ANOVA model of the *knowing-self* construct. *p*-values of less than the 0.05 significance level were highlighted in bold.

Predictor	Sum of Squares	d*f*	Mean Square	*F*	*p*-Value	Partial η^2^
(Intercept)	1269.983	1	1269.983	2679.013	0.000	0.973
Gender	3.478	2	1.739	3.668	**0.030**	0.089
Profession	0.284	2	0.142	0.299	0.742	0.008
Educational level	0.912	1	0.912	1.924	0.169	0.025
Professional experience	0.008	2	0.004	0.008	0.992	0.000
Error	35.554	75	0.474			

### Appendix A.4. Clarity of Beliefs and Values

An analysis of variance (ANOVA) was performed to determine the effect of the variables gender, profession, educational level, and professional experience on the construct *clarity of beliefs and values* (Table A4).

The analysis of the standardized residual diagnostic charts, residual Q–Q chart, and residual histogram (Figure A4) did not show any apparent violation of the model’s assumptions of normality and homoscedasticity.

**Table A4:** Results of the ANOVA model of the *clarity of beliefs and values* construct. *p*-values of less than the 0.05 significance level were highlighted in bold.

Predictor	Sum of Squares	d*f*	Mean Square	*F*	*p*-Value	Partial η^2^
(Intercept)	1113.45	1	1113.45	3204.91	0.000	0.977
Gender	0.707	2	0.353	1.017	0.366	0.026
Profession	0.712	2	0.356	1.025	0.365	0.027
Educational level	0.247	1	0.247	0.711	0.402	0.009
Professional experience	1.045	2	0.522	1.503	0.229	0.039
Error	26.056	75	0.347			

### Appendix A.5. Developed Interpersonal Skills

In the construct *developed interpersonal skills*, it was necessary to resort to the Box–Cox transformation, since there were considerable deviations from the assumptions of heteroscedasticity and normality of the residues. With the new variable, the analysis of the standardized residuals, residual Q–Q diagnostic plots, and histogram of the residuals (Figure A5) did not show clear violations of the model’s assumptions of normality and homoscedasticity.

An analysis of variance (ANOVA) was performed on the transformed variable to determine the effect of the different variables of gender, profession, educational level, and professional experience on the construct of *developed interpersonal skills* (Table A5).

**Table A5:** Results of the ANOVA model of the *developed interpersonal skills* construct. *p*-values of less than the 0.05 significance level were highlighted in bold.

Predictor	Sum of Squares	d*f*	Mean Square	*F*	*p*-Value	Partial η^2^
(Intercept)	0.000	1	0.000	0.000	0.000	0.000
Gender	0.100	2	0.050	0.050	0.951	0.001
Profession	4.454	2	2.227	2.221	0.116	0.056
Educational level	0.009	1	0.009	0.009	0.927	0.000
Professional experience	3.328	2	1.664	1.659	0.197	0.052
Error	75.215	75	1.003			

### Appendix A.6. Shared Decision-Making Systems

An analysis of variance (ANOVA) was performed to determine the effect of the variables gender, profession, educational level, and professional experience on the construct of *shared decision-making systems* (Table A6).

The analysis of the standardized residual diagnostic charts, residual Q–Q chart, and residual histogram (Figure A6) did not show any apparent violation of the model’s assumptions of normality and homoscedasticity.

**Table A6:** Results of the ANOVA model of the *shared decision-making systems* construct. *p*-values of less than the 0.05 significance level were highlighted in bold.

Predictor	Sum of Squares	d*f*	Mean Square	*F*	*p*-Value	Partial η^2^
(Intercept)	881.57	1	881.57	1467.33	0.000	0.951
Gender	1.255	2	0.627	1.044	0.357	0.027
Profession	6.465	2	3.233	5.381	**0.007**	0.125
Educational level	0.026	1	0.026	0.044	0.835	0.001
Professional experience	1.065	2	0.532	0.886	0.416	0.023
Error	45.060	75	0.601			

### Appendix A.7. The Physical Environment

An analysis of variance (ANOVA) was performed to determine the effect of the variables gender, profession, educational level, and professional experience on the construct of *the physical environment* (Table A7).

The analysis of the standardized residual diagnostic charts, residual Q–Q chart, and residual histogram (Figure A7) did not show any apparent violation of the model’s assumptions of normality and homoscedasticity.

**Table A7:** Results of the ANOVA model of *the physical environment* construct. *p*-values of less than the 0.05 significance level were highlighted in bold.

Predictor	Sum of Squares	d*f*	Mean Square	*F*	*p*-Value	Partial η^2^
(Intercept)	1060.32	1	1063.32	2337.13	0.000	0.969
Gender	3.608	2	1.804	3.634	**0.031**	0.088
Profession	0.430	2	0.215	0.433	0.650	0.011
Educational level	0.148	1	0.148	0.298	0.587	0.004
Professional experience	0.361	2	0.180	0.363	0.697	0.010
Error	37.235	75	0.496			

### Appendix A.8. Appropriate Skill Mix

An analysis of variance (ANOVA) was performed to determine the effect of the variables gender, profession, educational level, and professional experience on the construct ***a****ppropriate skill mix* (Table A8).

The analysis of the standardized residual diagnostic charts, residual Q–Q chart, and residual histogram (Figure A8) did not show any apparent violation of the model’s assumptions of normality and homoscedasticity.

**Table A8:** Results of the ANOVA model of *appropriate skill mix* construct. *p*-values of less than the 0.05 significance level were highlighted in bold.

Predictor	Sum of Squares	d*f*	Mean Square	*F*	*p*-Value	Partial η^2^
(Intercept)	1341.37	1	1341.37	4895.82	0.000	0.985
Gender	0.097	2	0.049	0.117	0.838	0.005
Profession	0.455	2	0.228	0.831	0.440	0.022
Educational level	0.782	1	0.782	2.853	0.095	0.037
Professional experience	0.264	2	0.132	0.482	0.619	0.013
Error	25.549	75	0.274			

### Appendix A.9. Effective Staff Relationships

An analysis of variance (ANOVA) was performed to determine the effect of the variables gender, profession, educational level, and professional experience on the construct of *effective staff relationships* (Table A9).

The analysis of the standardized residual diagnostic charts, residual Q–Q chart, and residual histogram (Figure A9) did not show any apparent violation of the model’s assumptions of normality and homoscedasticity.

**Table A9:** Results of the ANOVA model of *effective staff relationships* construct. *p*-values of less than the 0.05 significance level were highlighted in bold.

Predictor	Sum of Squares	d*f*	Mean Square	*F*	*p*-Value	Partial η2
(Intercept)	1086.748	1	1086.748	1814.498	0.000	0.960
Gender	0.796	2	0.398	0.664	0.518	0.017
Profession	1.614	2	0.807	1.348	0.266	0.035
Educational level	0.034	1	0.034	0.057	0.812	0.001
Professional experience	0.495	2	0.248	0.413	0.663	0.011
Error	44.919	75	0.599			

### Appendix A.10. Power Sharing

An analysis of variance (ANOVA) was performed to determine the effect of the variables gender, profession, educational level, and professional experience on the construct *power sharing* (Table A10).

The analysis of the standardized residual diagnostic charts, residual Q–Q chart, and residual histogram (Figure A10) did not show any apparent violation of the model’s assumptions of normality and homoscedasticity.

**Table A10:** Results of the ANOVA model of *power sharing* construct. *p*-values of less than the 0.05 significance level were highlighted in bold.

Predictor	Sum of Squares	d*f*	Mean Square	*F*	*p*-Value	Partial η^2^
(Intercept)	1027.28	1	1027.28	2271.30	0.000	0.968
Gender	0.031	2	0.016	0.035	0.966	0.001
Profession	0.960	2	0.480	1.061	0.351	0.028
Educational level	0.489	1	0.489	1.082	0.302	0.014
Professional experience	0.473	2	0.237	0.523	0.595	0.014
Error	33.921	75	0.452			

### Appendix A.11. Potential for Innovation and Risk-Taking

An analysis of variance (ANOVA) was performed to determine the effect of the variables gender, profession, educational level, and professional experience on the construct *potential for innovation and risk-taking* (Table A11).

The analysis of the standardized residual diagnostic charts, residual Q–Q chart, and residual histogram (Figure A11) did not show any apparent violation of the model’s assumptions of normality and homoscedasticity.

**Table A11:** Results of the ANOVA model of *potential for innovation and risk-taking* construct. *p*-values of less than the 0.05 significance level were highlighted in bold.

Predictor	Sum of Squares	d*f*	Mean Square	*F*	*p*-Value	Partial η^2^
(Intercept)	1067.53	1	1067.53	2443.85	0.000	0.970
Gender	0.167	2	0.084	0.191	0.826	0.005
Profession	0.382	2	0.191	0.437	0.647	0.012
Educational level	0.096	1	0.096	0.219	0.641	0.003
Professional experience	0.797	2	0.398	0.912	0.406	0.024
Error	33.909	75	0.437			

### Appendix A.12. Supportive Organization System

An analysis of variance (ANOVA) was performed to determine the effect of the variables gender, profession, educational level, and professional experience on the construct *supportive organization system* (Table A12).

The analysis of the standardized residual diagnostic charts, residual Q–Q chart, and residual histogram (Figure A12) did not show any apparent violation of the model’s assumptions of normality and homoscedasticity.

**Table A12:** Results of the ANOVA model of *supportive organization system* construct. *p*-values of less than the 0.05 significance level were highlighted in bold.

Predictor	Sum of Squares	d*f*	Mean Square	*F*	*p*-Value	Partial η^2^
(Intercept)	789.59	1	789.59	1166.88	0.000	0.940
Gender	0.421	2	0.210	0.311	0.734	0.008
Profession	0.084	2	0.042	0.062	0.940	0.002
Educational level	0.132	1	0.132	0.194	0.661	0.003
Professional experience	0.426	2	0.213	0.314	0.731	0.008
Error	50.750	75	0.677			

### Appendix A.13. Working with the Person’s Beliefs and Values

An analysis of variance (ANOVA) was performed to determine the effect of the variables gender, profession, educational level, and professional experience on the construct *working with the person’s beliefs and values* (Table A13).

The analysis of the standardized residual diagnostic charts, residual Q–Q chart, and residual histogram (Figure A13) did not show any apparent violations of the model’s assumptions of normality and homoscedasticity.

**Table A13:** Results of the ANOVA model of *working with the person’s beliefs and values* construct. *p*-values of less than the 0.05 significance level were highlighted in bold.

Predictor	Sum of Squares	d*f*	Mean Square	*F*	*p*-Value	Partial η^2^
(Intercept)	1360.193	1	1360.193	4209.643	0.000	0.982
Gender	1.567	2	0.783	2.425	0.095	0.061
Profession	0.350	2	0.175	0.542	0.584	0.014
Educational level	0.023	1	0.023	0.072	0.789	0.001
Professional experience	0.522	2	0.261	0.808	0.450	0.021
Error	24.234	75	0.323			

### Appendix A.14. Sharing Decision Making

An analysis of variance (ANOVA) was performed to determine the effect of the variables gender, profession, educational level, and professional experience on the construct of *sharing decision making* (Table A14).

The analysis of the standardized residual diagnostic charts, residual Q–Q chart, and residual histogram (Figure A14) did not show clear violations of the model’s assumptions of normality and homoscedasticity.

**Table A14:** Results of the ANOVA model of *sharing decision making* construct. *p*-values of less than the 0.05 significance level were highlighted in bold.

Predictor	Sum of Squares	d*f*	Mean Square	*F*	*p*-Value	Partial η^2^
(Intercept)	1272.590	1	1272.590	2454.582	0.000	0.970
Gender	2.113	2	1.057	2.038	0.137	0.052
Profession	0.001	2	0.001	0.001	0.999	0.000
Educational level	0.010	1	0.010	0.020	0.888	0.000
Professional experience	2.435	2	1.217	2.348	0.103	0.059
Error	38.884	75	0.518			

### Appendix A.15. Engaging Authentically

An analysis of variance (ANOVA) was performed to determine the effect of the variables gender, profession, educational level, and professional experience on the construct of *engaging authentically* (Table A15).

The analysis of the standardized residual diagnostic charts, residual Q–Q chart, and residual histogram (Figure A15) did not show clear violations of the model’s assumptions of normality and homoscedasticity.

**Table A15:** Results of the ANOVA model of *engaging authentically* construct. *p*-values of less than the 0.05 significance level were highlighted in bold.

Predictor	Sum of Squares	d*f*	Mean Square	*F*	*p*-Value	Partial η^2^
(Intercept)	1445.142	1	1445.142	5134.736	0.000	0.986
Gender	0.187	2	0.093	0.332	0.719	0.009
Profession	0.060	2	0.030	0.106	0.899	0.003
Educational level	0.309	1	0.309	1.097	0.298	0.014
Professional experience	0.075	2	0.037	0.133	0.876	0.004
Error	21.108	75	0.281			

### Appendix A.16. Being Sympathetically Present

An analysis of variance (ANOVA) was performed to determine the effect of the variables gender, profession, educational level, and professional experience on the construct of *being sympathetically present* (Table A16).

The analysis of the standardized residual diagnostic charts, residual Q–Q chart, and residual histogram (Figure A16) did not show clear violations of the model’s assumptions of normality and homoscedasticity.

**Table A16:** Results of the ANOVA model of *being sympathetically present* construct. *p*-values of less than the 0.05 significance level were highlighted in bold.

Predictor	Sum of Squares	d*f*	Mean Square	*F*	*p*-Value	Partial η^2^
(Intercept)	1376.434	1	1376.434	3910.046	0.000	0.981
Gender	1.555	2	0.777	2.209	0.117	0.056
Profession	0.391	2	0.196	0.556	0.576	0.015
Educational level	0.152	1	0.152	0.432	0.513	0.006
Professional experience	0.505	2	0.252	0.717	0.492	0.019
Error	26.402	75	0.352			

### Appendix A.17. Working Holistically

An analysis of variance (ANOVA) was performed to determine the effect of the variables gender, profession, educational level, and professional experience on the construct of *working holistically* (Table A17).

The analysis of the standardized residual diagnostic charts, residual Q–Q chart, and residual histogram (Figure A17) did not show clear violations of the model’s assumptions of normality and homoscedasticity.

**Table A17:** Results of the ANOVA model of *working holistically* construct. *p*-values of less than the 0.05 significance level were highlighted in bold.

Predictor	Sum of Squares	d*f*	Mean Square	*F*	*p*-Value	Partial η^2^
(Intercept)	1481.531	1	1481.531	3734.196	0.000	0.980
Gender	0.728	2	0.364	0.918	0.404	0.024
Profession	0.330	2	0.165	0.416	0.661	0.011
Educational level	0.139	1	0.139	0.351	0.555	0.005
Professional experience	0.489	2	0.245	0.616	0.543	0.016
Error	29.756	75	0.397			

## Appendix B

The correlation between the domains that composed the PCPI-S was verified (Table A18). There was a significant correlation in all domains, showing to be stronger between the *prerequisites* and *person-centered processes* (*p*-value = 0.417), followed by the *prerequisites* and *practice environment* (*p*-value = 0.392) and, finally, *the practice environment* and *person-centered processes* (*p*-value = 0.383).

**Table A18:** Correlation between the domains of the PCPI-S.

Correlation				
		Prerequisites	The Practice Environment	Person-Centered Processes
Prerequisites	Pearson correlation	1		
	Sig. (two-tailed)			
	N	83		
The practice environment	Pearson correlation	0.392 **	1	
	Sig. (two-tailed)	0.000		
	N	83	83	
Person-centered processes	Pearson correlation	0.417 **	0.383 **	1
	Sig. (two-tailed)	0.000	0.000	
	N	83	83	

** Correlation was significant at the 0.01 level (two-tailed).
